# Modified cyclodialysis suturing with sodium hyaluronate for traumatic choroidal avulsion and cyclodialysis: a retrospective study

**DOI:** 10.1186/s40942-025-00796-w

**Published:** 2026-01-10

**Authors:** Xueyong Zhang, Yao Xu, Wenji Xu, Tianxu Feng, Zhenyu Li, Yiming Zhu, Yao Chen, Siqi Xiong

**Affiliations:** 1 Eye Center of Xiangya Hospital, Central South University, No.87 Xiangya Street, Kaifu District, Changsha, Hunan 410008 China; 2 Hunan Key Laboratory of Ophthalmology, Central South University, Changsha, China; 3 National Clinical Research Center for Geriatric Disorders, Xiangya Hospital, Central South University, Changsha, China

**Keywords:** Choroid, Ciliary body, Trauma

## Abstract

**Background:**

Traumatic choroidal avulsion combined with cyclodialysis cleft is a severe form of ocular trauma that frequently leads to profound vision loss and persistent hypotony. Conventional surgical approaches often fail to achieve stable anatomical reattachment. This study aimed to evaluate the efficacy and safety of a modified cyclodialysis suturing technique combined with sodium hyaluronate injection for repairing traumatic choroidal avulsion and cyclodialysis cleft.

**Methods:**

This retrospective study included 12 patients (mean age: 47.17 ± 15.84 years) with choroidal avulsion and ciliary body detachment who underwent vitrectomy, sodium hyaluronate injection into the vitreous cavity prior to cyclodialysis repair to facilitate choroidal repositioning, and modified internal cyclopexy. Main outcome measures included anatomical reattachment rate of the choroid and ciliary body (assessed by ultrasound biomicroscopy), best-corrected visual acuity, intraocular pressure, and surgical complications. Follow-up was at least 6 months.

**Results:**

Anatomical success (complete reattachment of choroid and closure of cyclodialysis cleft) was achieved in 10 of 12 eyes (83.3%). The best-corrected visual acuity improved significantly from 2.90 ± 0.04 logMAR preoperatively to 2.60 ± 0.13 at 6 months (*p* = 0.019). Intraocular pressure increased from 8.92 ± 1.68 mmHg to 12.25 ± 2.96 mmHg at 6 months (*p* = 0.003). No serious intraoperative or postoperative complications occurred.

**Conclusion:**

The modified cyclodialysis suturing technique combined with sodium hyaluronate injection is a safe and effective approach for repairing traumatic choroidal avulsion and cyclodialysis cleft, offering high anatomical success and significant improvement in visual acuity and intraocular pressure. This method provides a valuable surgical option for this challenging condition.

**Supplementary Information:**

The online version contains supplementary material available at 10.1186/s40942-025-00796-w.

## Background

Choroidal avulsion is a severe detachment of the choroid from the sclera, usually due to major open globe injuries. This can cause rupture of blood vessels, leading to significant bleeding and persistent low eye pressure [1]. It often occurs with other complications like retinal detachment, making repair challenging. In some cases, both the choroid and ciliary body detach [1]. Choroidal avulsion is the most serious traumatic choroidal injury, with over 92% of cases having poor outcomes, often resulting in phthisis bulbi and being difficult to repair even with advanced surgical techniques [2].

Treating choroidal avulsion is challenging due to the difficulty in reattaching and stabilizing the delicate choroid, along with managing retinal detachment and other complications. It remains a significant challenge in eye surgery, with limited literature available. Recent advancements in surgical techniques, such as the use of fibrin glue or suture methods, have been reported to improve outcomes in cases of choroidal avulsion [1, 3–5]. Fibrin glue is limited in complex cases due to its need for a dry environment. Trans-scleral suture fixation (TSS) and intraocular suturing (IOS) are used to reattach avulsed choroids, with IOS preferred for significant anterior choroid detachment. Studies indicate a high success rate in complete reattachment with suturing [1]. However, these methods’ effectiveness is uncertain in severe ocular trauma with choroidal avulsion where the tissue self-adheres.

This study introduces a novel approach combining sodium hyaluronate with a modified single-arm cyclopexy suture technique. Our innovation addresses the unique challenge of choroidal-ciliary body avulsion as a single unit, a scenario inadequately managed by prior methods.

## Materials and method

### Aim, design and setting of the study

This retrospective study sought to assess the efficacy and safety of a modified cyclodialysis suturing technique in conjunction with sodium hyaluronate injection for the repair of traumatic choroidal avulsion with an associated cyclodialysis cleft. The study involved a review of medical records of patients who received treatment for choroidal avulsion resulting from severe ocular trauma at the Ophthalmology Department of Xiangya Hospital, Central South University, between June 2021 and November 2023. The study design did not include a control group or randomization. Ethical approval was obtained from the hospital’s Ethics Committee, in compliance with the Declaration of Helsinki. Informed written consent was secured from all participants prior to their inclusion in the study.

The study involved patients with choroidal avulsion and ciliary body detachment due to severe eye injuries, also showing signs of anterior or posterior segment damage such as iris injury, cataract, lens dislocation, vitreous hemorrhage, retinal detachment, and various hemorrhages. Treated at Xiangya Hospital, these patients received sclera and/or cornea repair within 24 h and a 72-hour course of intravenous antibiotics. During secondary vitrectomy, a modified cyclodialysis suture technique with sodium hyaluronate and other procedures was performed as needed. Exclusion criteria included patients over 80 years of age, those with uveitis or endophthalmitis, and those with less than six months of follow-up. Ultimately, 12 patients were enrolled in the study.

### Clinical evaluation

Data collected before surgery included patient variables like eye history, age, gender, intraocular pressure (IOP), best-corrected visual acuity (BCVA), ultrasound biomicroscopy (UBM) and B-scan ultrasonography. Vision levels were standardized, with counting fingers vision equated to 1.9 logMAR and hand motions vision to 2.3 logMAR [6], light perception vision to 2.7 logMAR, and no light perception to 3.0 logMAR [7]. The intraocular pressure was assessed three times with a pneumotonometer (CT-800 computerized tonometer, Topcon Ltd., Tokyo, Japan), and the average value was noted. B-scan ultrasound was performed to assess the vitreous cavity, retina, and choroid. The UBM (Model SW-3200 L; Tianjin Suowei Electronic Technology Co., Ltd., Tianjin, China) was used to evaluate the size of the cyclodialysis cleft and the state of the iris injury. Patients were followed for at least six months post-surgery, with assessments of BCVA, IOP, retinal and choroid reattachment rates, and postoperative complications.

### Surgical procedure

Before surgery, a detailed assessment of the eye’s anterior (iris, lens, ciliary body) and posterior segments (vitreous, retina, choroid) was performed. Levofloxacin eye drops were applied to the surgical eye six times daily for three days. Dr. Siqi Xiong, an experienced surgeon, conducted all surgeries under general or retrobulbar anesthesia. For two patients with severe corneal opacification, a temporary keratoprosthesis (TKP) was used at the start of surgery to view the posterior segment. The removed cornea was stored in sodium hyaluronate. A balanced salt solution was injected into the vitreous to raise IOP, and any fluid or blood in the suprachoroidal space was drained via sclerotomy.

**Fig. 1:**
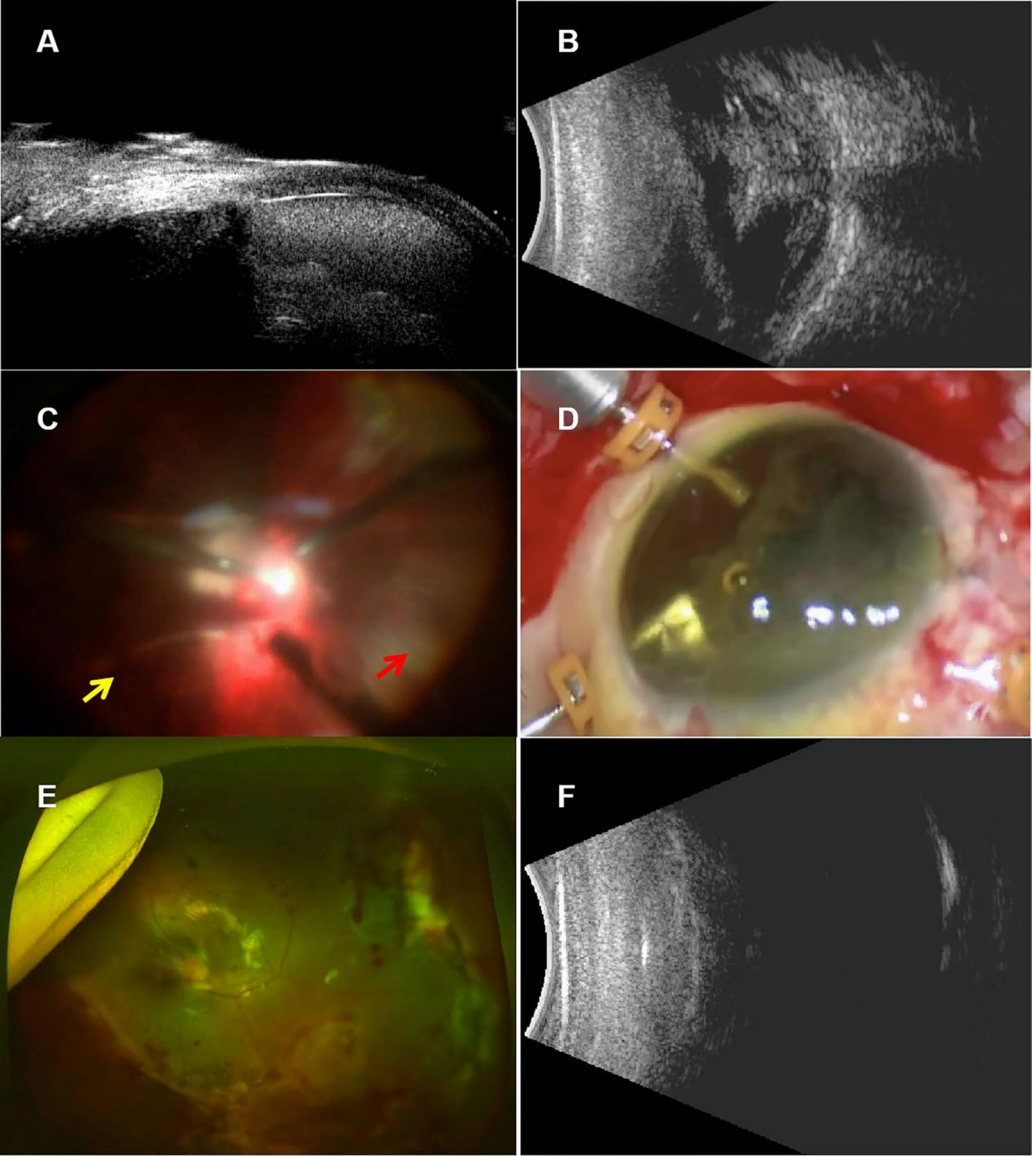
A modified single suture technique was employed in a patient presenting with traumatic cyclodialysis resulting from a zone III ruptured globe injury. (**A**) Preoperative ultrasound biomicroscopy (UBM) analysis revealed traumatic cyclodialysis accompanied by hyphema. (**B**) Preoperative B-scan ultrasonography identified the presence of vitreous hemorrhage. (**C**) Intraoperative photographs illustrated the avulsion of the choroid-ciliary body from the 12 o’clock to the 6 o’clock position, with the detached retina incarcerated into the scleral wounds. The yellow arrow denotes the location of the avulsed choroid, while the red arrow highlights the exposed sclera. (**D**) Intraoperative photographs illustrated the attached choroid after infusion with sodium hyaluronate. (**E**) Postoperative fundus photography and (**F**) B-scan ultrasonography demonstrated reattachment of the retina and choroid, supported by silicone oil tamponade

Insert the anterior chamber infusion through the limbus, making limbal incisions at the 2 o’clock and 10 o’clock positions. Vitrectomy cutter was used to remove vitreous, intraocular hemorrhage, and other opacities. Forceps were used to meticulously separate the uveal tissues caught in the wound, and 3 ml of sodium hyaluronate (Iviz^@^, Bausch + Lomb) was injected to reattach the ciliary body and choroid to the sclera. A conjunctival incision was made at the limbus, extending one clock hour beyond each end of the cyclodialysis cleft. A 10 − 0 polypropylene suture with a straight needle was inserted through the sclera, 2 mm behind the limbus on one side of the cleft. A 29-gauge needle guided the straight needle through the ciliary body and out at the opposite limbus incision. The straight needle was then reinserted through the same incision, guided out about 1 clock hour from the initial entry. The ciliary body was reattached with the suture, and the knots were secured in the sclera. This process was repeated until the cleft was fully repaired [8]. During a 3-port, 23-gauge transconjunctival scleral incision, a phacoemulsification tip removed hyaluronic acid from the vitreous cavity. Subretinal membranes were peeled, and a retinectomy released the retinal funnel from the scleral wound. Perfluorooctane was used to flatten the retina, and retinal photocoagulation treated breaks or the retinectomy edge. After a fluid-air exchange, silicone oil (Oxane 5700, Bausch + Lomb) filled the vitreous cavity (Fig. 1 and supplementary video). In the first two TKP cases, the TKP was removed post-surgery, and the autologous cornea was secured with 10 − 0 nylon sutures. TKP provided visibility for severe retinal and corneal injuries, as penetrating keratoplasty wasn’t performed due to the absence of a corneal donor.

Patients were advised to keep their heads stable for at least 12 h daily for four weeks post-surgery to aid retinal and choroid positioning. They received topical corticosteroids and antibiotics to prevent infections and reduce inflammation. Postoperative exams and data collection followed the clinical evaluation protocol.

### Statistical analyses

All data were analyzed using SPSS (version 19.0, Chicago, IL, USA). Descriptive statistics were employed to summarize the basic characteristics, including means ± standard deviations for continuous variables and frequencies (proportions) for categorical variables. The student t test was utilized to compare preoperative and postoperative data. A p-value of less than 0.05 was considered statistically significant.

## Results

### Characteristics of patient injuries before surgery

The study recruited 12 patients (12 eyes) with complete avulsion of the choroid and ciliary body. The cohort comprised 9 men and 3 women, with a mean age of 47.17 ± 15.84 years (range: 5–60 years). All patients were monitored for a minimum of 6 months (range: 6–18 months). The injuries were all due to open globe trauma, with 66.7% (8/12) resulting from rupture and 33.3% (4/12) from penetrating injuries. Zone 3 (extending more than 5 mm posterior to the limbus) was the most frequently affected area, involving 11 eyes (91.7%), while Zone 1 (cornea and limbus) was affected in one eye (8.3%). The ruptured globes were sutured within 24 h, and secondary vitrectomy surgeries were conducted on average 14.67 ± 4.54 days post-injury (range: 6–22 days). Further details of these preoperative characteristics are provided in Table 1.

**Table 1:** Basic characteristics of the enrolled patients

Objectives	Patients enrolled (*n* = 12)
Age, mean ± SD	47.17 ± 15.84
Gender, n (%)	
Male	9(75%)
Female	3(25%)
Type of injury, n (%)	
Open globe Injury	12(100%)
Blunt trauma	0(0%)
Traumatic zone, n (%)	
I II	1(8%) 0(0%)
III	11(92%)
Surgery time interval, mean ± SD (days)	14.67 ± 4.54
Hours of avulsed choroid	5.83 ± 2.12

SD, standard deviation; n, number; Surgery time interval, time interval between the surgery and the occurrence of trauma

### The degree of patient injury and postoperative anatomical recovery

The extent of choroidal avulsion was assessed during vitrectomy surgery following the removal of both suprachoroidal and vitreous hemorrhages. All eyes presented with additional severe traumatic complications, including corneal wound or opacity (7/12, 58.3%), iris injury (9/12, 75%), cyclodialysis cleft (12/12, 100%), hyphema (12/12, 100%), cataract (2/12, 16.7%), lens dislocation (2/12, 16.7%), lens extrusion (10/12, 83.3%), vitreous hemorrhage (12/12, 100.0%), retinal detachment (12/12, 100.0%), suprachoroidal hemorrhage (12/12, 100.0%), and subretinal hemorrhage (12/12, 100.0%). All enrolled patients underwent 23-gauge pars plana vitrectomy (PPV), and additional therapeutic interventions, including temporary keratoprosthesis, lensectomy, phacoemulsification, cyclopexy, retinectomy, and intraocular tamponade, were implemented as summarized in Table 2.

The mean extent of choroidal avulsion was 5.83 ± 2.13 clock hours(range: 2–10 clock hours). In nine eyes, the extent of the choroidal avulsion was larger than or equal to six clock hours(9/12, 75.0%). After surgery, the successful rate of cyclodialysis repair was 100% as confirmed by UBM. The reattachment of avulsed choriod was observed in 10 patients (83.3%) as confirmed by B-scan ultrasonography and fundus photography (Fig. 1). And retinal attachment was observed in 100.0% of patients.

**Table 2:** Ocular characteristics and surgical interventions

Ocular characteristics	Patients enrolled (*n* = 12)
Cornea wound/opacity, n (%)	7(58.3%)
Iris injury, n (%)	9(75.0%)
Cyclodialysis cleft, n (%)	12(100.0%)
Hyphema, n (%)	12(100.0%)
Lens extrusion, n (%)	10(83.3%)
Lens dislocation, n (%)	2(16.7%)
Vitreous hemorrhage, n (%)	12(100.0%)
Retina detachment, n (%)	12(100.0%)
Supra choroidal haemorrhage, n (%)	12(100.0%)
Temporary keratoprosthesis, n (%)	2 (16.7%)
Lensectomy/Phacoemulsification, n (%)	2(16.7%)
Cyclodialysis cleft repair, n (%)	12 (100.0%)
Iridodialysis repair, n (%)	9(75.0%)
Retinectomy, n (%)	12 (100.0%)
Silicone oil, n (%)	12(100.0%)

### Postoperative ocular function recovery status

**Fig. 2:**
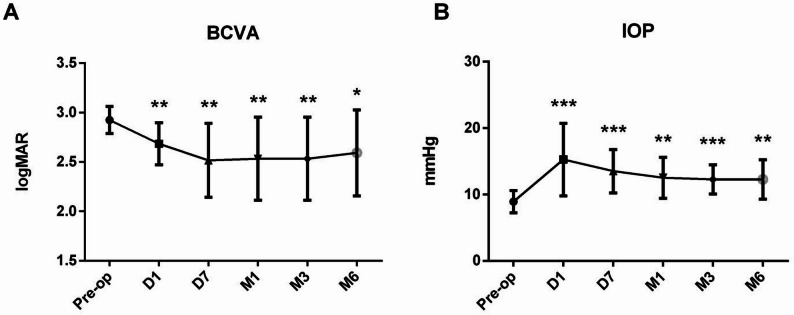
The BCVA and IOP of the patients. **A**. BCVA demonstrated a statistically significant improvement at 1 day (D1), 7 days (D7), 1 month (M1), 3 months (M3), and 6 months (M6) postoperatively, compared to the preoperative BCVA (*n* = 12). **B**. Intraocular pressure (IOP) exhibited a statistically significant increase at D1, D7, M1, M3, and M6 in comparison to the preoperative IOP (*n* = 12). Abbreviations: Preop: pre-operation; D1: 1 day after cyclopexy; D7: 1 week after surgery; M1: 1 month after surgery; M3: 3 months after surgery; M6: 6 months after surgery. (*P < 0.05, **P < 0.01, ***P < 0.001)

The median preoperative BCVA, measured as the logarithm of the minimum angle of resolution, was 2.90 ± 0.04, with nine eyes exhibiting no light perception (75.0%). A statistically significant improvement in BCVA was observed at each postoperative follow-up interval, including day 1 (BCVA = 2.72 ± 0.06, *p* = 0.004), day 7 (BCVA = 2.52 ± 0.11, *p* = 0.002), 1 month (BCVA = 2.53 ± 0.12, *p* = 0.006), 3 months (BCVA = 2.53 ± 0.12, *p* = 0.006), and 6 months (BCVA = 2.60 ± 0.13, *p* = 0.019). Additionally, compared to the preoperative IOP of 8.92 ± 1.68 mmHg, significant postoperative improvements were observed at day 1 (IOP = 15.25 ± 5.46 mmHg, *p* < 0.001), day 7 (IOP = 13.50 ± 3.26 mmHg, *p* < 0.001), 1 month (IOP = 12.50 ± 3.09 mmHg, *p* = 0.002), 3 months (IOP = 12.25 ± 2.18 mmHg, *p* < 0.001), and 6 months (IOP = 12.25 ± 2.96 mmHg, *p* = 0.003) postoperatively (Fig. 2).

### Incidence of postoperative complications

**Fig. 3:**
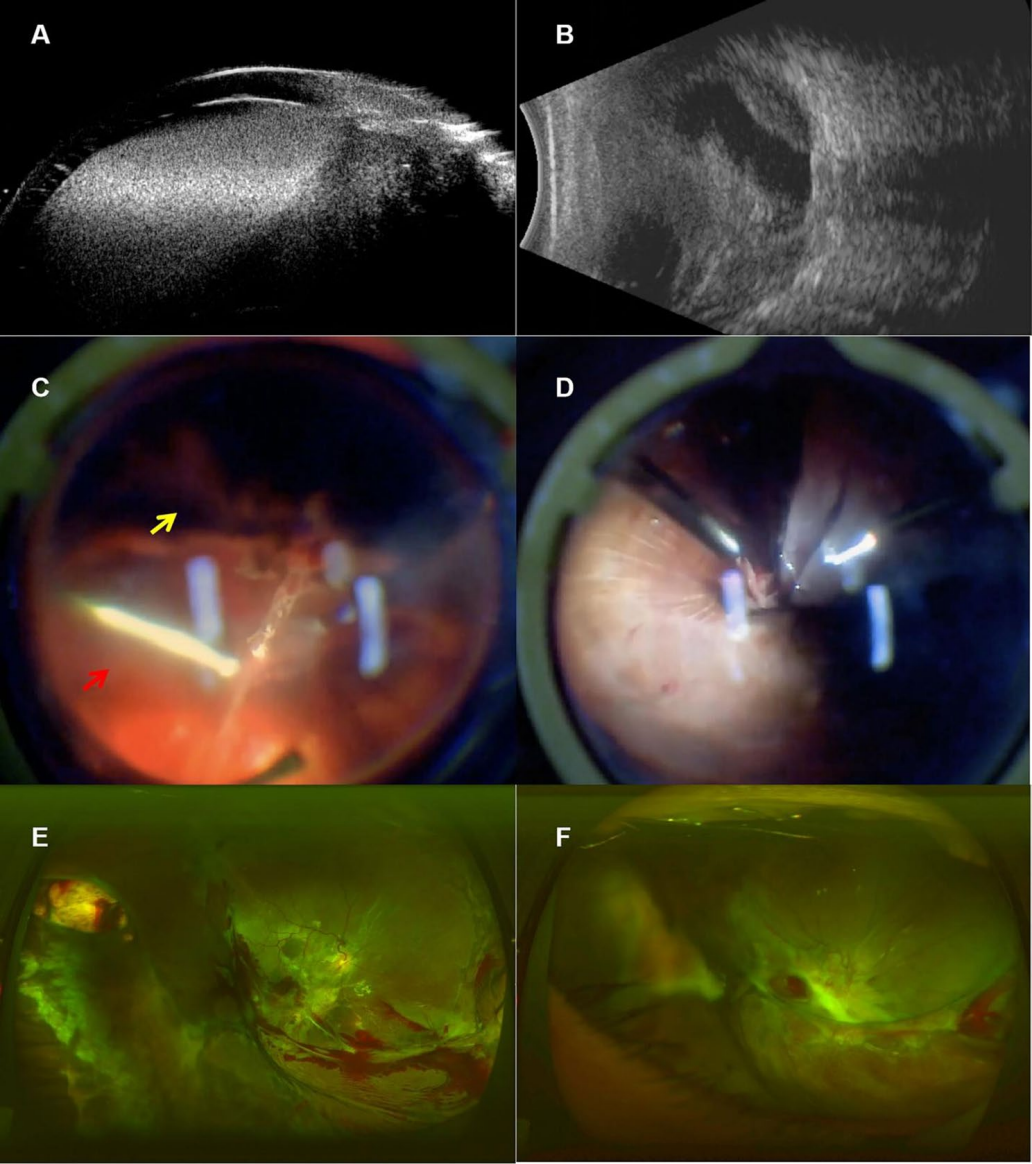
The patient with choroidal avulsion and cyclodialysis underwent a modified single suture technique and vitrectomy. One month postoperatively, a choroidal hole was identified, necessitating the transplantation of an amniotic membrane. (**A**) Preoperative ultrasound biomicroscopy revealed traumatic cyclodialysis accompanied by hyphema. (**B**) Preoperative B-scan ultrasonography identified vitreous hemorrhage, suprachoroidal hemorrhage, and choroidal detachment. (**C**) Intraoperative photographs demonstrated avulsion of the choroid-ciliary body from the 3 o’clock to the 11 o’clock position. The yellow arrow denotes the location of the avulsed choroid, while the red arrow highlights the exposed sclera.(**D**) Intraoperative images demonstrated the adherence of the choroid following sodium hyaluronate infusion, accompanied by a funnel-shaped total retinal detachment that was incarcerated within the scleral scar. (**E**) Postoperative fundus photography at one month showed retinal reattachment with silicone oil tamponade, a choroidal hole at the 9 o’clock position near the ora serrata, and suprachoroidal migration of silicone oil. (**F**) The choroidal hole was successfully healed following the transplantation of the amnion

Postoperative complications observed in the study included choroidal hole (*n* = 1, 8.3%), fungal keratitis (*n* = 1, 8.3%), sympathetic ophthalmia (*n* = 1, 8.3%), leukoma (*n* = 2, 16.67%), and silicone oil dependency (*n* = 12, 100%). To address the choroidal hole, amnion transplantation was employed as a sealing technique (Fig. 3). In two cases, silicone oil removal was attempted 1 to 4 months following vitrectomy; however, these cases necessitated re-application of silicone oil tamponade due to the occurrence of ocular hypotension. Postoperative UBM was performed in 10 of 12 patients and confirmed closure of the cyclodialysis cleft and reattachment of the ciliary body in all examined cases (Supplementary Material 1).

## Discussion

Various surgical interventions have been explored to address choroidal avulsion. One reported case involved the injection of medical fibrin glue into the suprachoroidal cavity following air-fluid exchange, facilitating adhesion between the choroid and sclera [3]. The use of fibrin glue, however, is limited to non-aqueous environments post-air-fluid exchange [3]. In instances of severe choroidal avulsion accompanied by retinal incarceration, it is imperative to repair the choroid prior to addressing retinal detachment. Under such complex conditions, fibrin glue proves ineffective. Ma J et al. described a transscleral suture fixation technique involving a full-thickness scleral incision [4]. Nonetheless, this suturing approach may elevate the risk of uveal exposure and subsequent development of sympathetic ophthalmia postoperatively. Chen HJ et al. introduced innovative suturing techniques for the management of choroidal avulsion, specifically trans-scleral mattress suturing and intraocular suturing, achieving choroidal reattachment in 91.7% of cases [1]. However, these procedures can extend beyond four hours and present significant challenges for less experienced surgeons. Furthermore, the feasibility of these techniques may be limited in patients with extensive choroidal avulsion or pre-existing adhesions. In contrast, our modified technique offers the following advantages: (1) injection of sodium hyaluronate into the vitreous cavity to gently reposition the avulsed choroid without excessive manipulation; (2) direct visualization and mattress suturing under chandelier illumination, achieving higher rates of complete choroidal reattachment than traditional external cyclopexy; and (3) reduced risk of choroidal incarceration or further trauma. These modifications address the challenges of tissue self-adhesion failure in severe trauma, leading to normalized intraocular pressure under silicone oil tamponade and partial visual recovery in this retrospective cohort.

Severe mechanical ocular trauma can lead to ocular decompression and scleral expansion, while extensive choroidal hemorrhage increases pressure in the suprachoroidal space, causing separation of the choroid and ciliary body from the sclera. When accompanied by vortex vein rupture, the detached choroid and ciliary body become more mobile. The torn choroid may then become entrapped within the scleral wound, adhering to it and trapping the retina between the avulsed choroid, resulting in a characteristic ‘sandwich’ appearance. In such cases, the critical step in restoring ocular integrity is the reattachment of the choroid and ciliary body to the sclera, which is essential for the successful repair of other damaged ocular structures. The distinctive histological structure of the choroid contributes to its reduced elasticity compared to the retina, complicating reattachment efforts.

Perfluorocarbon liquids (PFCLs) are commonly used to aid retinal reattachment but can migrate due to low viscosity, especially in severe choroidal avulsion [9]. In contrast, sodium hyaluronate, a viscoelastic gel, offers high surface tension and viscosity, making it ideal for maintaining surgical space [10]. It can be easily removed during vitrectomy and is used to separate and reattach the avulsed choroid and ciliary body to the sclera [11]. The high surface tension and viscosity of sodium hyaluronate generate mechanical forces that aid in this process. This step is critical for the subsequent repair of the detached ciliary body using the modified single-armed suture technique, as previously reported [11]. Our study shows that using the modified cyclodialysis suture technique with sodium hyaluronate effectively repairs traumatic choroidal avulsion, achieving full choroidal reattachment and notable visual improvements. However, two patients had partial detachment due to delayed surgeries, causing choroidal fibrosis and stiffness.

Despite achieving complete choroidal and retinal reattachment with normalized intraocular pressure under silicone oil tamponade, all patients in this series remained silicone oil-dependent throughout the follow-up period to prevent recurrent hypotony and potential phthisis bulbi. This finding aligns with previous reports on severe traumatic choroidal avulsion, where most cases required permanent silicone oil tamponade [1]. The maintenance of normal intraocular pressure under silicone oil suggests adequate aqueous humor secretion by the reattached ciliary body. However, the long-term outcomes remain to be observed, as silicone oil removal was only attempted in two cases and resulted in hypotony necessitating re-injection. Potential mechanisms for hypotony following silicone oil removal may include incomplete functional recovery of the ciliary body or persistent dysfunction due to extensive choroidal exposure and fibrosis. In addition, the already torn vortex vein and posterior ciliary artery could not be anastomosed, complicating the reconstruction of choroidal circulation and further contributing to silicone oil dependence in these eyes. These findings highlight the need for longer-term follow-up studies and cautious management of silicone oil removal in such complex ocular trauma cases.

Previous studies have suggested that the development of choroidal hole following vitrectomy for ocular trauma may be associated with choroidal scar contraction or the structural vulnerability at the anterior-posterior uveal junction, where extraocular muscle contraction and adhesion commonly occur [12]. However, choroidal hole typically manifest after a postoperative interval. In our study, we observed intraoperative choroidal hole formation, which may be attributed to pre-existing choroidal fibrosis and partial choroidal repositioning.

Our modified cyclodialysis suture technique with sodium hyaluronate shows promise, but several limitations exist. The diversity in cases, such as varying injury mechanisms and complications, restricts the generalizability of our results. Due to the rarity of choroidal avulsion, recruiting larger cohorts is challenging, necessitating future multicenter studies for validation. The retrospective study design may also introduce selection bias and incomplete data, weakening our conclusions. Prospective or randomized controlled trials are needed to minimize bias and establish causality. Addressing these limitations in future research will enhance the clinical relevance of our findings.

## Conclusion

This study underscores the significance of early intervention and the necessity for innovative techniques in managing severe ocular trauma. The incorporation of sodium hyaluronate facilitated the reattachment process by safeguarding delicate tissues during manipulation, potentially accounting for the higher success rate compared to traditional methods.

## Supplementary Information

Below is the link to the electronic supplementary material.

Supplementary Material 1

Supplementary Material 2

## Data Availability

All data generated or analysed during this study are included in this published article.

## Abbreviations

BCVA: Best-corrected visual acuity
IOP: Intraocular pressure
IOS: Intraocular suturing
PFCLs: Perfluorocarbon liquids
PPV: Pars plana vitrectomy
TKP: Temporary keratoprosthesis
TSS: Trans-scleral suture fixation
UBM: Ultrasound biomicroscopy
