# Supplementary material for: Modified cyclodialysis suturing with sodium hyaluronate for traumatic choroidal avulsion and cyclodialysis: a retrospective study
Source: Int J Retina Vitreous. 2026 Jan 10;12:25. doi: 10.1186/s40942-025-00796-w (PMC12874734; doi:10.1186/s40942-025-00796-w)
Supplement: Supplementary file 1 — Supplementary Material 1 [file 40942_2025_796_MOESM1_ESM.docx]

Patient #1


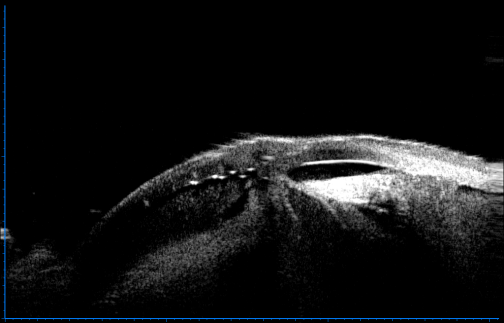

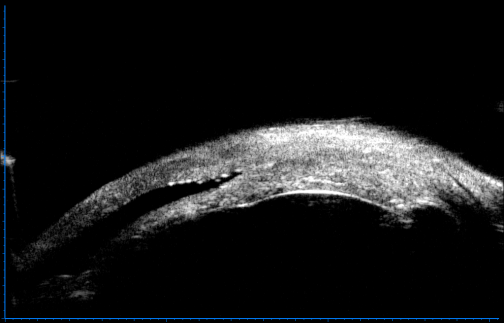


Before surgery After surgery

Patient #2


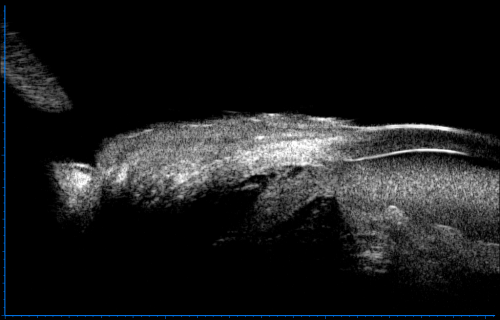

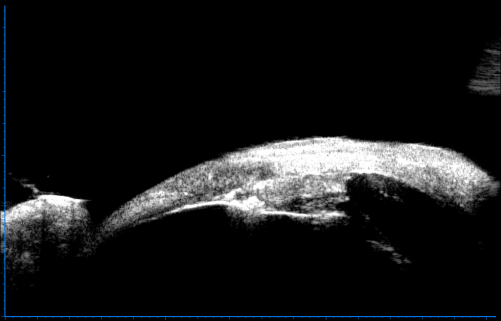


Before surgery After surgery

Patient #3


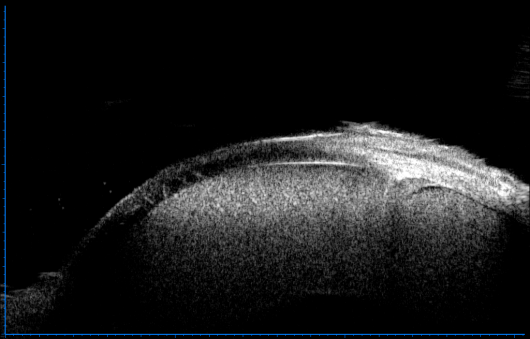

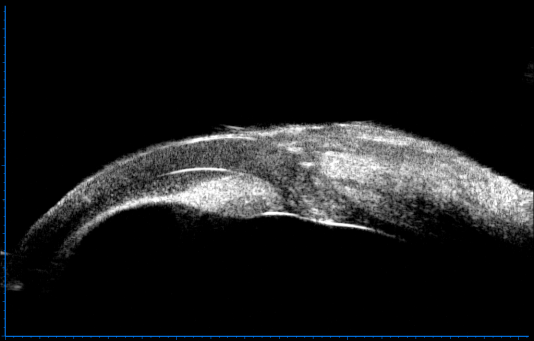


Before surgery After surgery

Patient #4


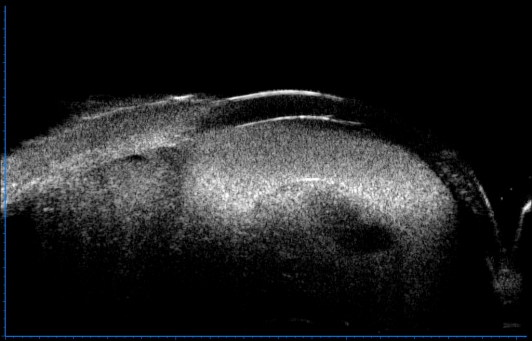

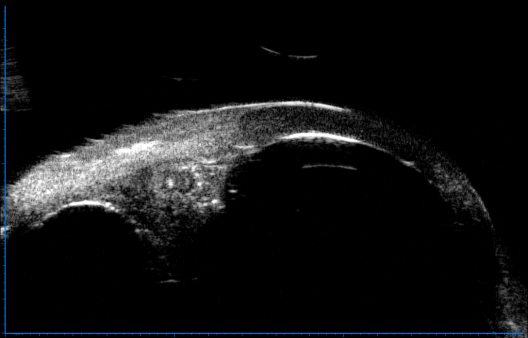


Before surgery After surgery

Patient #5


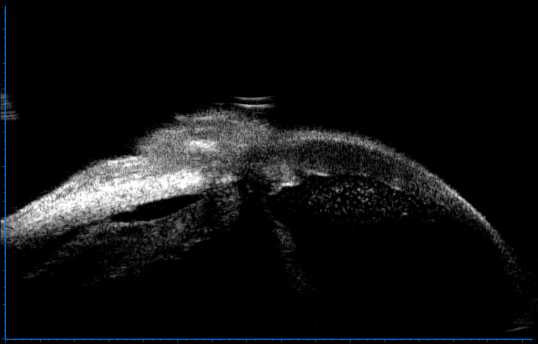

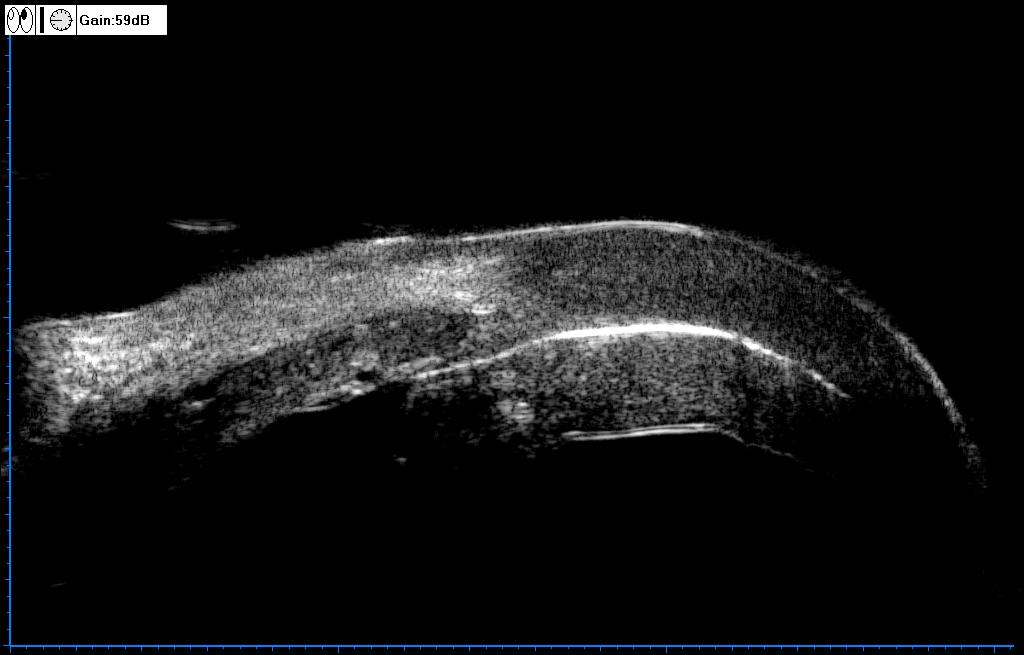


Before surgery After surgery

Patient #6


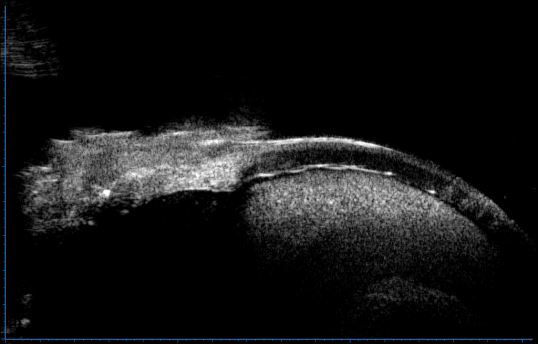

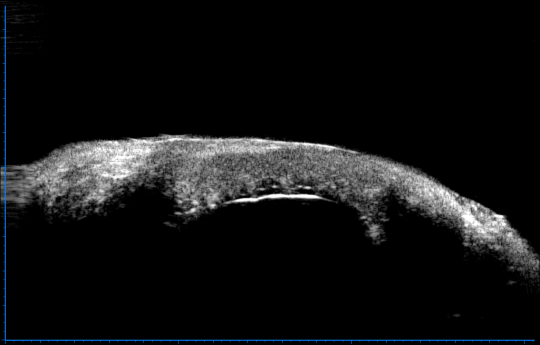


Before surgery After surgery

Patient #7


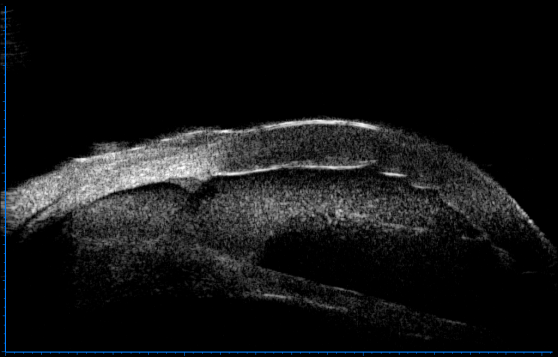

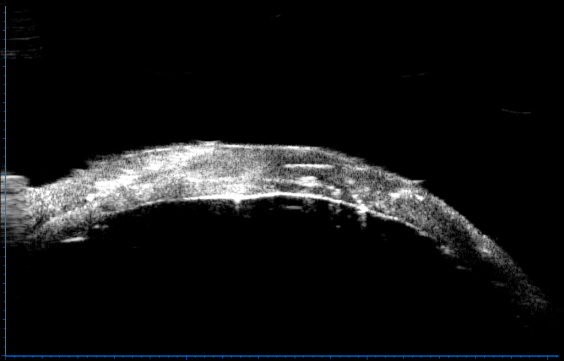


Before surgery After surgery

Patient #8


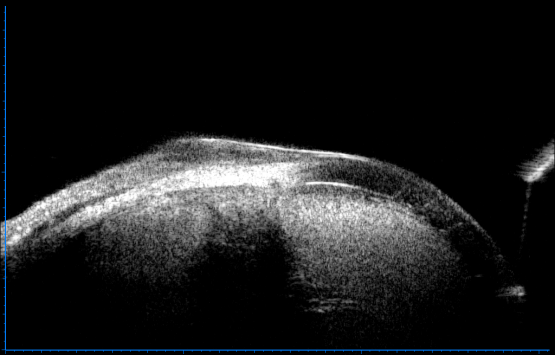

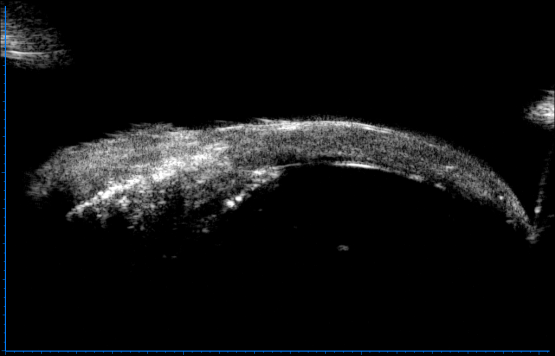


Before surgery After surgery

Patient #9


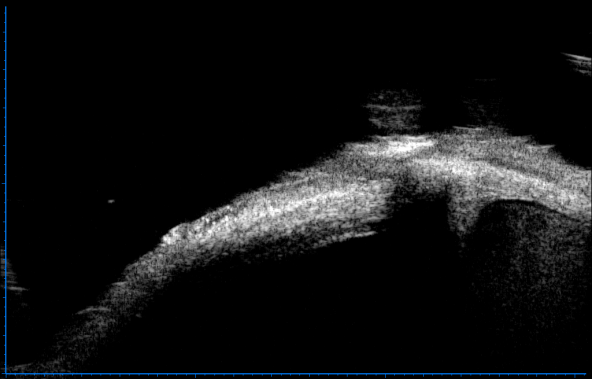

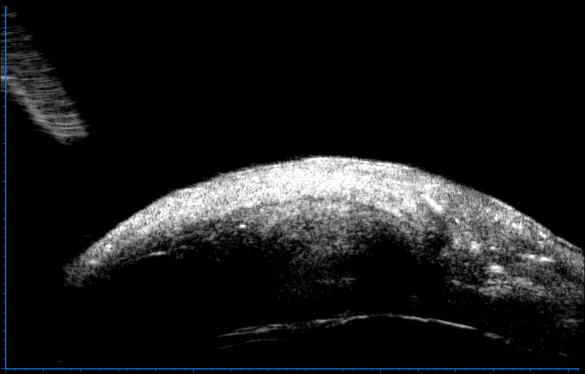


Before surgery After surgery

Patient #10


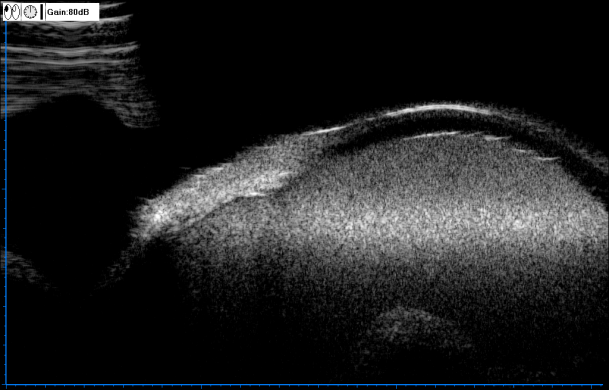

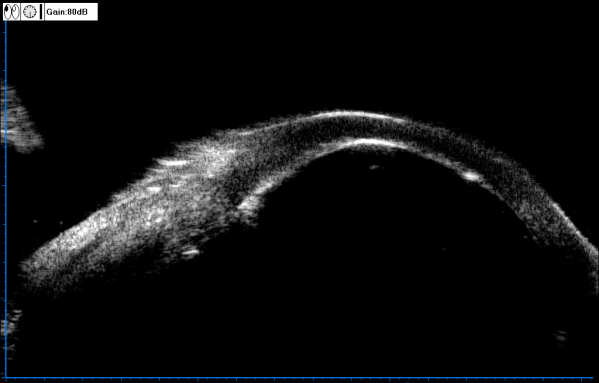


Before surgery After surgery
